# Using a multi-omics approach to explore potential associations with rumen content and serum of cows with different milk production levels based on genomic predicted transmitting ability for milk and phenotypic milk production

**DOI:** 10.1371/journal.pone.0305674

**Published:** 2024-07-18

**Authors:** Anay D. Ravelo, Peter Ferm, Yue Guo, Bobwealth O. Omontese, Paul S. Morley, Chi Chen, Noelle R. Noyes, Luciano S. Caixeta

**Affiliations:** 1 Department of Veterinary Population Medicine, University of Minnesota, Saint Paul, Minnesota, United States of America; 2 Department of Food Science and Nutrition, University of Minnesota, Saint Paul, Minnesota, United States of America; 3 Veterinary Education, Research, and Outreach Program, Texas A&M University, Canyon, Texas, United States of America; University of Illinois, UNITED STATES

## Abstract

This study aims to compare rumen microbiome and metabolites between second lactation dairy cows in the 75^th^ percentile (n = 12; 57.2 ± 5.08 kg/d) of production according to genomic predicted transmitting ability for milk (GPTAM) and their counterparts in the 25^th^ percentile (n = 12; 47.2 ± 8.61 kg/d). It was hypothesized that the metagenome and metabolome would differ between production levels. Cows were matched by days in milk (DIM), sire, occurrence of disease, and days open in previous lactation. For an additional comparison, the cows were also divided by phenotype into high (n = 6; 61.3 ± 2.8 kg/d), medium (n = 10; 55 ± 1.2 kg/d), and low (n = 8; 41.9 ± 5.6 kg/d) based on their milk production. Samples were collected 65 ± 14 DIM. Rumen content was collected using an oro-gastric tube and serum samples were collected from the coccygeal vessels. High-resolution liquid chromatography-mass spectrometry (LC-MS) was used for rumen and serum metabolite profiling. Shotgun metagenomics was used for rumen microbiome profiling. Microbiome sample richness and diversity were used to determine alpha and Bray-Curtis dissimilarity index was used to estimate beta diversity. Differences in metabolites were determined using t-tests or ANOVA. Pearson correlations were used to consider associations between serum metabolites and milk production. There was no evidence of a difference in rumen metabolites or microbial communities by GPTAM or phenotype. Cows in the phenotypic low group had greater serum acetate to propionate ratio and acetate proportion compared to the cows in the phenotypic medium group. Likewise, serum propionate proportion was greater in the medium compared to the low phenotypic group. Serum acetate, butyrate, and propionate concentrations had a weak positive correlation with milk production. When investigating associations between rumen environment and milk production, future studies must consider the impact of the ruminal epithelium absorption and post-absorption processes in relation to milk production.

## Introduction

Various factors can affect milk production of dairy cows throughout their lactation period, such as feed ingredients, digestion and absorption of nutrients within the digestive tract, variations in individual metabolism, disease events, animal welfare, environmental factors, genetics and uptake of nutrients to the mammary gland [1]. Besides these factors, the influences of ruminal microbial communities on milk composition, feed efficiency and methane production of cattle have also been documented [2, 3]. As the sources of these influences, the rumen microbiome and metabolome are affected by nutritional factors, stressors, and physiological status [4], but their correlations with milk production, as well as their contribution, have seldom been examined.

Cows have a symbiotic relationship with the microbial communities in their rumen. Using ingested feed as the substrates for fermentation, rumen microbes meet the nutritional requirements of dairy cows through the production of various bioavailable metabolites that are absorbed for energy and nutrient metabolism [5]. The rumen microbiome is composed of bacteria, archaea, protozoa, fungi, and viruses [4]. These populations allow ruminants the advantage of being able to turn indigestible plant mass into energy and protein [6]. Microbial communities ferment feed substrates into metabolites that can be absorbed by the host. The rumen metabolome consists of the metabolites that are found in ruminal fluid [7]. These metabolites can be end products of microbial fermentation such as short chain fatty acids (SCFA). Some studies have considered the individual effects of the microbiome and metabolome of dairy cows with different levels of milk production [8, 9]. These studies observed that there are different populations and metabolite markers at different levels of milk production [8, 9]. Few have considered the effects of the microbiome and metabolome together [10–12]. These studies observed that there were differences in the relative abundance of some bacteria as well as metabolic differences in cows with different milk production levels. Understanding associations between the rumen microbiome and metabolome, as well as associations to host metabolome, with milk production could help to modulate and enhance milk production outcomes in the future.

Traits such as milk, components, somatic cells, etc could be used to compare animals. A trait can be assigned an estimate which would be its predicted transmitting ability (PTA) [13]. The genomic PTA for milk could be used to compare high versus low producing animals. The GPTAM considers an average of zero and animals that have a negative value are predicted to perform below the average and animals with a positive value are predicted to perform above average for a breed. As milk production in cows continues to increase it is important to explore associations with GPTAM and the rumen environment and host genome to better understand what factors could be contributing to differences in milk production between animals.

Thus, the purpose of this study was to identify potential rumen microbial populations and metabolites, as well as serum metabolites, that are associated with different milk production groups as identified by their genomic predicted transmitting ability for milk. A secondary explorative objective was to identify potential rumen microbial populations and metabolites, as well as serum metabolites, that are associated with high, medium, and low milk production as identified by phenotypic milk production. It was hypothesized that the microbiome and metabolite profile of the rumen content, as well as that of the serum, will be more favorable for milk production in high producing cows compared to low producing dairy cows.

## Materials and methods

The University of Minnesota Institutional Animal Use and Care Committee approved all the procedures for animal care and handling required for this experiment (protocol number: 1806-36010A).

### Experimental design

**Fig 1:**
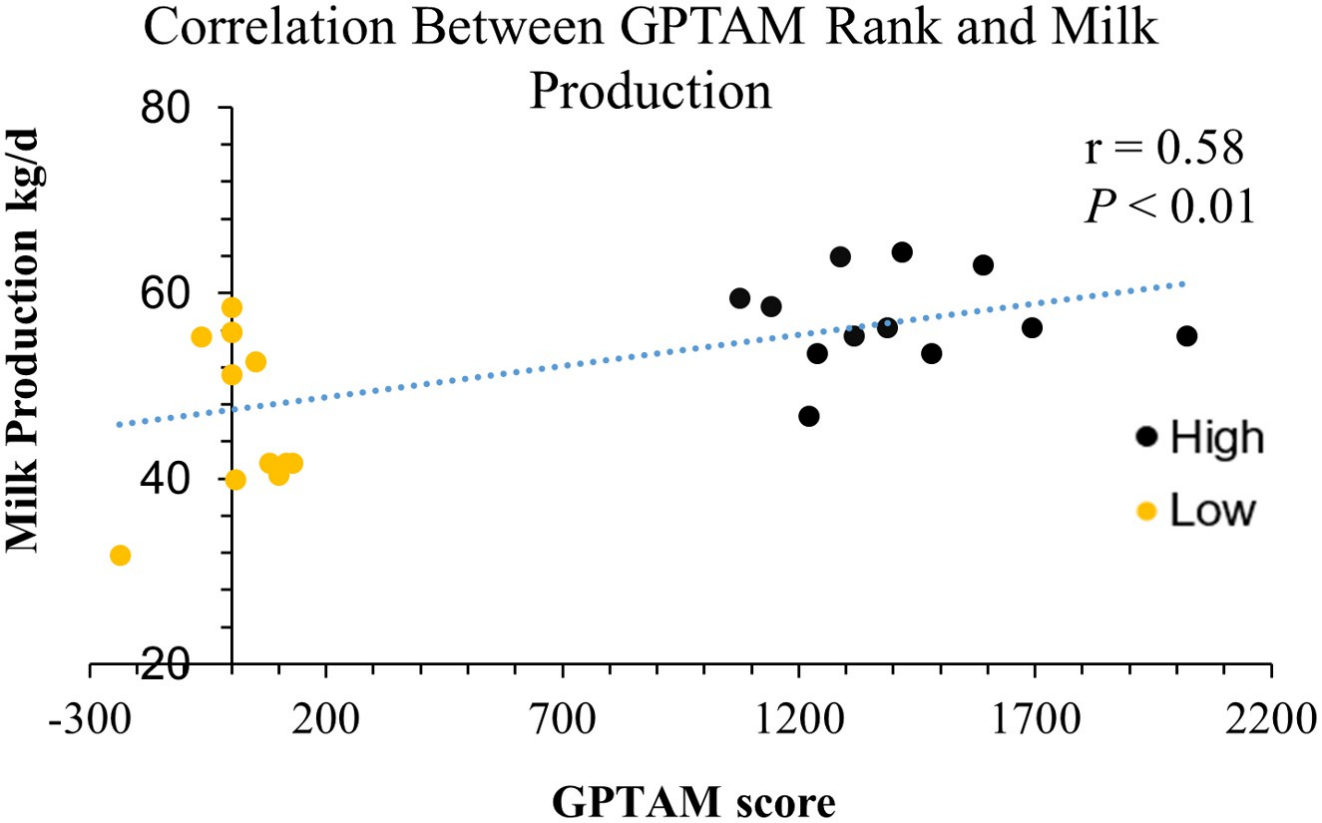
Pearson correlations for GTPAM and milk production. The Pearson correlations for the genomic predicted transmitting ability for milk (GPTAM) in relation to milk production in dairy cows.

This study was conducted in a commercial dairy farm with 1,194 cows (474 (40%) primiparous; 342 (29%) second lactation, and 378 (32%) third lactation or greater). A 12 matched-pairs (n = 24) study was performed to characterize and compare the ruminal microbiota and metabolites, as well as the serum metabolites, of high (n = 12; 57.2 kg/d) and low (n = 12; 47.2 kg/d) milk producing dairy cows based on their genomic predicted transmitting ability for milk (GPTAM). Samples were collected from October 2018 to February 2019. Cows were blocked by days in milk (DIM), sire, occurrence of disease during the sampling lactation, and days open in previous lactation. The grouping was determined by dividing cows into percentiles based GPTAM. Cows in ≥75^th^ percentile (high producing) were match to cows in the ≤25^th^ percentile (low producing; Table 1; Fig 1). The GPTAM data was available because the farm had previously genetically tested their cows using a commercially available genetic test for management reasons unrelated to this experiment (CLARIFIDE, Zoetis Inc.). Only second lactation cows between 60 and 90 DIM were eligible for enrollment as these cows are no longer growing, less likely to get sick compared to older cows, and producing a reasonable amount of milk. All cows were housed in free stall barns and fed a total mixed ration consisting of 55% forage and 45% concentrate. On a dry matter basis, the crude protein was 17.3%, acid detergent fiber was 19.7%, neutral detergent fiber was 31.9%, lignin was 2.7%, nonfiberous carbohydrates was 38.7%, starch was 27.1%, crude fat was 4.2%, ash was 7.95%, and total digestible nutrients was 73.0%, with a net energy for lactation of 1.71 Mcal/kg. The diet was formulated to meet the NRC nutrient requirement for lactating Holsteins according to farm conditions [14]. After enrollment based on GPTAM, cows were also divided based on their milk production (i.e., phenotype) into high (n = 6; 61.3 ± 2.8 kg/d), medium (n = 10; 55 ± 1.2 kg/d), and low (n = 8; 41.9 ± 5.6 kg/d) producing cows for a secondary analysis. This distribution was calculated based on the distribution of the milk production of all second lactation cows around 60–90 DIM in the herd at that time.

**Table 1:** Descriptive statistics of high versus low producing cows that were categorized by genomic predicted transmitting ability for milk (GPTAM) for comparison of serum metabolites and rumen content microbiome and metabolites.

Item	GPTAM^1^^,^^2^		*P*-value^3^
	High	Low	
GPTAM	1,406.3 (263.7)	14.8 (98.2)	<0.01
Body condition score^4^^,^^5^			0.27
2.5	1 (8.3)	0 (0.0)	
2.75	3 (25.0)	0 (0.0)	
3	4 (33.3)	3 (25.0)	
3.25	2 (16.7)	5 (41.7)	
3.5	1 (8.3)	3 (25.0)	
3.75	1 (8.3)	1 (8.3)	
Days in milk (DIM), d	63.2 (7.6)	66.8 (9.3)	0.30
Phenotype^5^^,^ ^6^			0.02
High	5 (41.7)	1 (8.3)	
Med	6 (50.0)	4 (33.3)	
Low	1 (8.3)	7 (58.3)	
Milk, kg/d	57.2 (5.1)	47.2 (8.6)	<0.01
Predicted total fat, kg	624.2 (176.5)	450.5 (62.9)	<0.01
Predicted total protein, kg	493.5 (121.1)	339.2 (36.8)	<0.01
305 day predicted milk yield, kg	15,968.3 (2348.1)	11,611.5 (1309.7)	<0.01
Previous lactation DIM, d	363.0 (58.5)	307.6 (13.0)	<0.01
Days dry, d	55.4 (4.8)	58.2 (9.9)	0.39
Health events^5^^,^^7^	8 (66.7)	7 (58.3)	1.00

^1^High n = 12 and low n = 12.

^2^Results presented as mean and standard deviation unless specified otherwise.

^3^*P*-value comparing the descriptive statistics for high versus low producing dairy cows.

^4^Body condition score was assessed using a 5-point scale with quarter point increments [15].

^5^Number and percentage of total.

^6^Phenotype based on observable milk production.

^7^Health events occurring in the study lactation prior to sample collection: mastitis, metritis, ketosis and off feed.

### Sample collection

Rumen content and blood samples were collected around 65 ± 14 days in milk to avoid differences in microbiome due to diet change. Rumen content was collected using an oro-gastric tube (Flora Rumen Scoop, Profs-Product) and the first collection of 50 mL of rumen content was discarded to avoid saliva contamination. The second collection of 50 mL of rumen content was collected and stored in 50 mL conical tubes. The tube was washed thoroughly between each sampling from cow to cow and autoclaved between samplings campaigns. Rumen content samples were put on ice and transported to the laboratory where they were stored in a -80°C freezer until further analysis.

Blood samples were collected from the coccygeal vessels into serum tubes and were kept on ice during transport to the laboratory to be spun down. Upon arrival to the lab, the serum tubes were centrifuged at 2,000 × *g* for 15 min. The supernatant was aliquoted into 2 microcentrifuge tubes and stored at -80°C until analysis.

### Sample preparation for LC-MS analysis

Rumen content samples were mixed with a 50% aqueous acetonitrile (ACN) using a 1:10 (*v*/*v*) ratio. The mixture was centrifuged at 16,000 × *g* for 10 min and the supernatant was collected for analysis. Serum samples were mixed with a 66% aqueous solution of ACN in a 1:20 (*v*/*v*) ratio. For detecting metabolites that contain a functional amino group, the samples were derivatized with dansyl chloride prior to the high-resolution liquid chromatography-mass spectrometry (LC-MS) analysis [16]. For detecting carboxylic acids, the samples were derivatized with 2-hydrazinoquinoline (HQ) prior to the LC-MS analysis (PMID: 24958262).

### LC-MS analysis (Targeted)

A 5 μL sample of the processed rumen content and serum were injected into an Acquity ultra-performance liquid chromatography (UPLC) system and separated in a BEH C18 column. For the amino acids (AA) in a 10-min run the mobile phase consisted of 0.1% formic acid in H_2_O and 0.1% formic acid in ACN with the MS detection mode as positive. For the SCFAs in a 10-min run the mobile phase consisted of 2 mM NH_4_OAc in water with 0.05% acetic acid and 2 mM NH_4_OAc in 95% ACN and 5% H_2_O with 0.05% acetic acid with the MS detection mode as positive. For accurate mass measurement and ion counting, the LC eluant was injected into a Xevo-G2-S QTOF mass spectrometry (Waters, Milford, MA, USA). For the positive-mode detection, the capillary voltage and cone voltage were maintained at 3kV and 30V, respectively. Argon was used as collision gas and nitrogen as both cone (50 L/h) and desolvation gas (600 L/h). The mass spectrometer was calibrated with sodium formate solution (range *m*/*z* 50–1000) for accurate mass measurement and monitored by the intermittent injection of the lock mass leucine encephalin ([M + H]+ = *m/z* 556.2771). MassLynx software (Waters) was used to acquire and process mass chromatograms and mass spectral data in a centroid format. Tandem MS (MS/MS) fragmentation with collision energies ranging from 15 to 45 eV was used for additional structural information. The concentrations of SCFA and AA were determined by calculating the ratio, the metabolite’s individual peak areas, and the peak area of the internal standard fitted with a standard curve using QuanLynx software (Waters).

### Extraction of rumen content for microbiome analysis

Rumen samples were thawed at room temperature and homogenized using a vortex. A 1 mL aliquot from the fluid was transferred to a microcentrifuge tube. The DNA extraction was performed using a QIAamp PowerFecal DNA kit (Qiagen, Germantown, Maryland, USA). Briefly, 0.25 g of rumen content was weighted and added to a bead tube with buffer and vortexed for lysing. Finally, the samples were processed through several centrifugation and filtration steps using buffers until the DNA was eluted. DNA concentration was assessed using a fluorimetric PicoGreen assay. DNA quality (A260/280 ratio) was assessed using a NanoDrop ND-1000 (Thermo Fisher Scientific, Waltham, Massachusetts, USA). One DNA aliquot was archived at -80°C and another was processed for sequencing.

#### Microbiome profiling

For a sample to move from DNA extraction to library preparation, it needed to have greater than 5 ng/μL of DNA. The 24 rumen content samples were prepared for sequencing at the University of Minnesota Genomics Center (UMGC, St. Paul, Minnesota, USA), using Illumina’s DNA Prep Sample Preparation Kit (Illumina, San Diego, California, USA) following manufacturer’s protocol. Adapters and primers were used from the Illumina DNA library kit and no sample or gene specific primers were used. The final library size distribution was validated using capillary electrophoresis and quantified using fluorimetry, and then loaded onto an Illumina NovaSeq 6000 S4 flow cell for 2x150bp paired-end sequencing.

#### Metagenome alignment and annotation

Minnesota Supercomputing Institute (MSI) serves were used for bioinformatics analysis. Metagenomes for each sample were processed using the AMR++ v2.0.0 pipeline [17]. Initially, fastq files were trimmed using trimmomatic v0.33 to remove adapter sequences and low-quality reads. The host genome was indexed, and the trimmed reads were aligned to the host genome using bwa v0.7.17 algorithms, bwa index and bwa mem, respectively. Then samtools was used to extract reads that did not map to the host genome. These unmapped reads were then converted back to fastq files using bedtools v2.27.1 and classified using kraken2 v2.0.7 (https://github.com/EnriqueDoster/bioinformatic-nextflow-pipelines/blob/master/main_AmrPlusPlus_v2_withKraken.nf) with a confidence flag of both zero, which is the default setting in kraken2, and 0.9. The database used for kraken2 was the standard Refseq database. The results for the metagenomic samples were then analyzed using python scripts with Python v3.8.3. Counts for strain-level classifications were aggregated to the species level and the entire taxonomic lineage of each feature was output into a matrix containing counts for each feature within each sample for downstream statistical analysis using R. The metagenome sequencing data was deposited into the NCBI SRA database (accession number PRJNA1006003).

### Statistical analysis

A total of 12 matched-pairs (n = 24) were enrolled in this study based on GPTAM. An exploratory analysis was conducted where the cows previously enrolled were separated by phenotypic milk production. At the time of study design and study enrollment, sample size calculations for studies using microbiome techniques were not standardized. However, other controlled studies in dairy cows have captured significant microbiome differences with as few as 5 individuals per group [18, 19]. Therefore, due to the exploratory nature of the project, the enrollment of 12 cows per group was considered to be sufficient for evaluating the primary research questions.

Metabolite concentrations and proportions of SCFA and AA in the rumen content and serum were compared using Student’s *t*-tests in R 4.2.2 (https://www.r-project.org/) for genotype. For the phenotype comparison, a one-way ANOVA was performed.

Analyses at the species level of metagenomic features were conducted in R 4.2.2. Both the unfiltered and filtered matrixes were considered, however, most results are present for the filtered matrix since the results and conclusions did not differ by confidence setting. Alpha diversity metrics for the bacterial communities were obtained using the “estimate_richness” function in the phyloseq package [20]. A student’s *t-*test and one-way ANOVA was used to compare the genotype and phenotype, respectively, richness (Chao1) and diversity (Shannon). The OTUs that had a relative abundance greater than 0.01% were retained for analysis. The counts were normalized using cumulative sum scaling from the metagenomeSeq package [21], and non-metric multidimensional scaling (NMDS) plots were created using the Bray-Curtis dissimilarity between samples, which was calculated from the vegan package [22]. Statistical comparison between the communities were conducted using the “adonis2” function in vegan and included the effect of genotype for one model and phenotype for the other. The differential abundance was determined using the “DESeq” function in phyloseq. The square root transformed OTU relative abundances were plotted in canonical correspondence analysis plot and the “envfit” function in vegan was used to fit the metabolite concentrations. The ggplot package was used to convert the plot into a ggplot. Pearson correlations were used to correlate milk production to rumen and serum metabolite concentrations. Results are discussed as precision of estimates using confidence intervals rather than in the context of statistical significance [23].

## Results

### Rumen content metabolome

Although the rumen content was analyzed for asparagine, citrulline, taurine, arginine, tryptophan, and glutamine, these amino acids were not included in any statistical analyses as they were quantified in a low number of samples, and the samples that present a concentration had the exact same concentration to 4 decimal points. Thus, their presentation was more probable to be lab error than a significant result.

Moreover, no evidence of a difference was observed in the concentrations or percentages of the SCFA (Table 2) or AA concentrations (Table 3) of the rumen content when comparing GPTAM high versus low producing cows. The percentage of methionine in the high producing group (0.1%; 95% CI: 0.04, 0.16) was less compared to the low producing group (0.2%; 95% CI: 0.14, 0.26; *P* = 0.10). Similarly, no evidence of differences was observed when comparing the concentrations in the different phenotype milk production groups (Tables 4 and 5).

**Table 2:** Comparisons of short chain fatty acids (SCFA) in the rumen content collected with a gastric tube of cows with high versus low milk production based on genomic predicted transmitting ability for milk (GPTAM).

Ruminal SCFA	GPTAM^1^		SEM	*P*-Value^2^
	High	Low		
Concentration (μM)				
Acetate	3138.5	3047.1	223.6	0.84
Propionate	857.9	887.0	100.8	0.89
Butyrate	4979.3	5011.8	308.3	0.96
Valerate	929.3	903.0	101.8	0.90
Isovalerate	942.2	921.4	104.5	0.92
A:P Ratio^3^	3.7	3.4	0.39	0.76
Total (μM)	10,847.1	10,770.3	717.6	0.97
Percentage (%)				
Acetate	27.8	30.5	2.47	0.45
Propionate	7.5	7.8	0.71	0.72
Butyrate	47.2	46.2	1.70	0.68
Valerate	8.7	7.6	0.76	0.33
Isovalerate	8.8	7.8	0.76	0.36

^1^High n = 12 and low n = 12.

^2^*P*-value of Student’s *t*-test.

^3^A:P Ratio: acetate to propionate ratio.

**Table 3:** Comparisons of amino acids (AA) in the rumen content collected with a gastric tube of cows with high versus low milk production based on genomic predicted transmitting ability for milk (GPTAM).

Ruminal AA	Concentration (μM)^1^		SEM	*P*-Value^3^	Percentage (%)^2^		SEM	*P*-Value^3^
	High	Low			High	Low		
Alanine	148.4	107.5	23.1	0.39	32.9	39.3	3.16	0.16
Aspartic Acid	20.5	13.5	5.51	0.54	2.5	1.9	0.76	0.60
Glutamic Acid	12.4	3.26	4.66	0.34	2.7	2.6	1.24	0.94
Glycine	57.6	39.6	10.5	0.40	11.8	10.7	0.71	0.31
Isoleucine/Leucine	15.8	9.43	3.54	0.38	2.0	1.6	0.42	0.44
Methionine	0.50	0.55	0.12	0.84	0.1	0.2	0.03	0.08
Phenylalanine	14.0	8.30	2.79	0.32	1.9	1.7	0.34	0.62
Proline	95.8	75.5	18.3	0.59	16.2	19.5	3.10	0.46
Serine	2.12	2.23	0.73	0.94	0.3	0.4	0.10	0.57
Threonine	11.6	7.70	2.66	0.48	1.5	1.4	0.32	0.88
Tyrosine	0.98	0.73	0.20	0.56	0.2	0.2	0.04	0.67
Valine	55.6	36.4	8.95	0.29	10.9	11.5	0.82	0.66
Lysine	1.37	0.58	0.28	0.16	0.2	0.2	0.04	0.88
Histidine	0.25	0.13	0.04	0.26	0.03	0.04	0.01	0.63
Ornithine	0.03	0.01	0.09	0.10	0.05	0.02	0.01	0.16
AABA^4^	0.43	0.11	8.15	0.30	10.4	8.8	1.28	0.42

^1^Concentrations and

^2^percentage of ruminal AA in high (n = 12) and low (n = 12) milk producing cows divided by genotype based on GPTAM.

^3^*P*-value of Student’s *t*-test.

^4^AABA = Alpha-aminobutyric acid.

**Table 4:** Comparisons of short chain fatty acids (SCFA) in the rumen content collected with a gastric tube of cows with high, medium, and low milk production based on phenotype.

Ruminal SCFA	Phenotype^1^			SEM	*P*-Value^2^
	High	Med	Low		
Concentration (μM)					
Acetate	3057.3	3,083.0	3,131.6	346.4	0.99
Propionate	861.0	838.7	923.3	156.1	0.94
Butyrate	4,899.5	5,155.0	4,868.4	477.6	0.92
Valerate	897.8	894.3	957.3	157.7	0.96
Isovalerate	910.7	911.7	972.8	161.8	0.97
A:P Ratio^3^	3.6	3.7	3.4	0.61	0.61
Total (μM)	10,626.3	10,882.7	10,853.3	1,111.6	0.99
Percentage (%)					
Acetate	27.8	28.0	31.7	3.13	0.62
Propionate	8.1	7.2	7.9	0.90	0.76
Butyrate	46.5	48.6	44.6	2.07	0.35
Valerate	8.8	8.0	7.9	0.98	0.83
Isovalerate	8.9	8.1	8.0	1.63	0.83

^1^Phenotype based on observable milk production: high n = 6, medium n = 10, low n = 8.

^2^*P*-value of one-way ANOVA.

^3^A:P Ratio: acetate to propionate ratio.

**Table 5:** Comparisons of amino acids (AA) in the rumen content collected with a gastric tube of cows with high, medium, and low milk production based on phenotype.

Ruminal AA	Concentration (μM)^1^			SEM	*P*-Value^3^	Percentage (%)^2^			SEM	*P*-Value^3^
	High	Med	Low			High	Med	Low		
Alanine	147.5	116.2	128.0	35.8	0.88	39.6	32.9	37.5	4.07	0.48
Aspartic Acid	16.3	15.3	19.7	8.53	0.94	1.8	2.1	2.5	0.97	0.90
Glutamic Acid	20.3	3.39	4.01	11.5	0.32	5.8	0.8	2.4	1.40	0.07
Glycine	55.7	45.0	47.8	16.2	0.93	10.9	11.8	10.8	0.92	0.67
Isoleucine/Leucine	15.2	11.4	12.2	5.49	0.92	1.6	2.0	1.7	0.54	0.83
Methionine	0.52	0.41	0.68	0.18	0.65	0.2	0.1	0.2	0.05	0.54
Phenylalanine	13.4	10.6	10.2	4.33	0.91	1.5	2.1	1.7	0.43	0.60
Proline	85.5	84.0	87.8	28.4	0.99	11.1	20.4	19.9	3.72	0.20
Serine	1.8	1.6	3.1	1.13	0.67	0.3	0.3	0.4	0.13	0.71
Threonine	10.7	8.8	9.9	4.12	0.96	1.3	1.6	1.5	0.41	0.90
Tyrosine	1.0	0.6	1.1	0.31	0.72	2.3	0.2	0.2	0.88	0.21
Valine	54.0	42.3	44.5	13.9	0.88	11.9	10.3	11.9	1.14	0.52
Lysine	1.3	1.0	0.7	0.43	0.77	0.3	0.2	0.1	0.06	0.39
Histidine	0.3	0.2	0.1	0.06	0.69	0.03	0.04	0.04	0.01	0.91
Ornithine	0.4	0.3	0.1	0.14	0.54	0.04	0.04	0.02	0.01	0.41
AABA^4^	47.8	36.5	36.5	12.6	0.85	11.4	8.7	9.3	1.69	0.52

^1^Concentrations and

^2^percentage of ruminal AA in high (n = 6), medium (n = 10) and low (n = 8) milk producing cows divided by phenotype based on observable milk production.

^3^*P*-value of one-way ANOVA.

^4^AABA = Alpha-aminobutyric acid.

### Serum metabolome

Although serum was analyzed for aspartic acid, this amino acid was not used in the statistical analysis due to the same reasoning presented above for the amino acids that were not included in the rumen content analysis. There was no evidence of a difference in the concentration or percentages of the SCFA (Table 6) or AA concentrations (Table 7) of the serum when comparing GPTAM high versus low producing cows. When comparing the results for phenotype, the acetate to propionate (A:P) ratio was greater in the low milk production group (5.3; 95% CI: 4.5, 6.0) compared to the medium milk production group (3.9; 95% CI: 3.2, 4.6; *P* = 0.02; Table 8). Additionally, when considering the percentage of the metabolites acetate and propionate, there was a greater proportion of acetate in the low milk production group samples (72.4%; 95% CI: 68.2, 76.6) compared to the medium milk production group samples (64.7%; 95% CI: 60.5, 68.9; *P* = 0.03). In addition, there was a greater proportion of propionate in the medium milk producing group samples (17.4%; 95% CI: 15.4, 19.4) when compared to the percentage in the low milk producing group (13.9%; 95% CI: 11.9, 15.9; *P* = 0.04). There were no differences observed in the AA concentration comparing the three phenotypic groups (Table 9).

**Table 6:** Comparisons of short chain fatty acids (SCFA) in the serum of cows with high versus low milk production based on genotype.

Serum SCFA	Genotype^1^		SEM	*P*-Value^2^
	High	Low		
Concentration (μM)				
Acetate	772.4	791.3	64.3	0.84
Propionate	197.9	180.3	29.1	0.67
Butyrate	103.0	90.7	16.0	0.59
Valerate	59.8	52.1	9.15	0.57
Isovalerate	41.9	36.5	6.37	0.56
A:P Ratio^3^	4.4	4.8	0.34	0.36
Total (μM)	1175.0	1150.9	112.9	0.88
Percentage (%)				
Acetate	67.1	69.7	1.94	0.34
Propionate	16.2	15.2	0.91	0.45
Butyrate	8.3	7.6	0.57	0.36
Valerate	5.0	4.4	0.39	0.34
Isovalerate	3.5	3.1	0.28	0.35

^1^Genotype based on GPTAM. High n = 12 and low n = 12.

^2^*P*-value of Student’s *t*-test.

^3^A:P Ratio: acetate to propionate ratio.

**Table 7:** Comparisons of amino acids (AA) in the serum of cows with high versus low milk production based on genomic predicted transmitting ability for milk (GPTAM).

Serum AA	Concentration (μM)^1^		SEM	*P*-Value^3^	Percentage (%)^2^		SEM	*P*-Value^3^
	High	Low			High	Low		
Alanine	108.5	114.1	5.89	0.51	14.7	14.3	0.53	0.76
Asparagine	0.6	0.7	0.10	0.45	0.07	0.08	0.01	0.49
Citrulline	0.7	0.7	0.10	0.74	0.10	0.08	0.01	0.33
Glutamic Acid	0.8	0.6	0.07	0.18	0.11	0.08	0.01	0.06
Glycine	273.5	302.1	30.3	0.52	35.6	37.9	2.44	0.51
Isoleucine/Leucine	14.6	12.9	2.06	0.58	1.9	1.7	0.26	0.45
Methionine	1.0	1.1	0.13	0.70	0.14	0.14	0.02	0.94
Phenylalanine	16.0	14.9	1.36	0.56	2.5	1.9	0.18	0.33
Proline	83.1	91.8	7.22	0.42	11.3	11.3	0.66	0.95
Serine	5.8	6.4	1.00	0.71	0.7	0.8	0.08	0.84
Taurine	0.9	0.8	0.12	0.41	0.1	0.1	0.02	0.26
Threonine	18.6	19.2	4.02	0.92	2.3	2.3	0.36	0.99
Tyrosine	5.9	4.0	1.41	0.38	0.8	0.5	0.14	0.20
Valine	150.7	145.3	10.3	0.71	20.5	18.7	1.48	0.39
Arginine	0.6	0.5	0.10	0.51	0.08	0.06	0.01	0.32
Lysine	0.7	0.7	0.17	0.92	0.09	0.09	0.02	0.87
Histidine	4.0	4.0	0.60	0.93	0.5	0.5	0.06	0.59
Tryptophan	2.0	2.1	0.29	0.81	0.3	0.3	0.03	0.98
Ornithine	0.1	0.2	0.05	0.17	0.01	0.02	0.01	0.21
GABA^4^	2.8	2.2	0.50	0.42	0.4	0.3	0.07	0.32
Glutamine	58.6	70.4	7.51	0.29	7.9	8.8	0.89	0.47

^1^Concentrations and

^2^percentage of serum AA in high (n = 12) and low (n = 12) milk producing cows divided by genotype based on GPTAM.

^3^*P*-value of Student’s *t*-test.

^4^GABA = Gamma-aminobutyric acid.

**Table 8:** Comparisons of short chain fatty acids (SCFA) in the serum of cows with high, medium, and low milk production based on phenotype.

Serum SCFA	Phenotype^1^			SEM	P-value^2^
	High	Med	Low		
Concentration (μM)					
Acetic Acid	855.8	798.2	706.1	79.1	0.45
Propionic Acid	185.4	234.6	135.1	33.5	0.10
Butyric Acid	96.9	120.2	67.7	18.7	0.13
Valeric Acid	59.1	69.1	37.0	10.4	0.09
Isovaleric Acid	41.4	48.4	26.0	7.29	0.09
A:P Ratio^3^	4.7^a^^b^	3.9^b^	5.3^a^	0.38	0.03
Total (μM)	1238.6	1270.5	971.9	134	0.23
Percentage (%)					
Acetate	69.3^a^^b^	64.7^b^	72.4^a^	2.16	0.04
Propionate	15.1^a^^b^	17.4^a^	13.9^b^	1.01	0.04
Butyrate	7.6	8.9	6.9	0.66	0.08
Valerate	4.8	5.3	4.0	0.47	0.14
Isovalerate	3.3	3.7	3.3	0.33	0.14

^1^Phenotype based on observable milk production: high n = 6, medium n = 10, low n = 8.

^2^*P*-value of one-way ANOVA.

^3^A:P Ratio: acetate to propionate ratio.

^a-b^Means within a row with different subscripts differ (P ≤ 0.05).

**Table 9:** Comparisons of amino acids (AA) in the serum of cows with high, medium, and low milk production based on phenotype.

Serum AA	Concentration (μM)^1^			SEM	*P*-Value^3^	Percentage (%)^2^			SEM	*P*-Value^3^
	High	Med	Low			High	Med	Low		
Alanine	115.5	110.4	109.3	7.54	0.85	14.9	14.8	14.1	0.68	0.66
Asparagine	0.6	0.7	0.6	0.12	0.81	0.07	0.09	0.08	0.01	0.53
Citrulline	0.8	0.6	0.7	0.13	0.66	0.10	0.08	0.09	0.01	0.69
Glutamic Acid	0.8	0.7	0.6	0.09	0.71	0.10	0.10	0.08	0.01	0.53
Glycine	333.5	272.0	273.2	38.2	0.49	41.6	35.2	35.2	3.00	0.29
Isoleucine/Leucine	11.3	15.1	13.8	2.62	0.62	1.5	2.0	1.8	0.33	0.65
Methionine	0.9	1.1	1.2	0.17	0.59	0.1	0.1	0.2	0.19	0.45
Phenylalanine	14.1	17.2	14.3	1.66	0.32	1.8	2.3	1.9	0.22	0.24
Proline	78.1	89.0	92.3	9.46	0.59	10.1	11.8	11.6	0.79	0.29
Serine	6.0	6.2	6.0	1.30	0.99	0.7	0.8	0.7	0.29	0.83
Taurine	0.9	0.8	0.8	0.16	0.95	0.12	0.11	0.11	0.02	0.26
Threonine	15.8	20.2	19.4	5.27	0.84	2.0	2.5	2.3	0.50	0.79
Tyrosine	3.0	6.5	4.5	1.85	0.40	0.4	0.8	0.5	0.19	0.27
Valine	134.3	149.2	156.9	12.8	0.51	17.6	19.8	20.9	1.86	0.50
Arginine	0.4	0.6	0.6	0.13	0.63	0.05	0.07	0.07	0.02	0.66
Lysine	0.6	0.8	0.7	0.22	0.72	0.07	0.10	0.08	0.02	0.44
Histidine	3.4	4.4	3.9	0.74	0.64	0.4	0.6	0.5	0.08	0.33
Tryptophan	1.9	2.0	2.1	0.36	0.89	0.3	0.3	0.3	0.03	0.93
Ornithine	0.1	0.2	0.2	0.07	0.68	0.01	0.02	0.02	0.01	0.65
GABA^4^	3.0	2.3	2.3	0.64	0.72	0.4	0.3	0.3	0.09	0.77
Glutamine	63.8	57.6	73.6	9.57	0.46	7.8	7.9	9.3	1.12	0.57

^1^Concentrations and

^2^percentage of serum AA in high (n = 6), medium (n = 10) and low (n = 8) milk producing cows divided by phenotype based on observable milk production.

^3^*P*-value of one-way ANOVA.

^4^GABA = Gamma-aminobutyric acid.

### Microbial communities

There was a total of 2,376,039,364 unmapped reads across all samples, with 327,090,238 mapped reads. Thus, 12.1% of reads were successfully aligned which is similar to that observed in other studies for rumen content [24]. From a total of 1,368,211,945 input reads across all samples, 14,768,103 or 1.1% of the reads were classified to a specific taxonomic group, which is comparable to the percentage classified in other studies [11]. For the taxa that were present at greater than 0.01% abundance with zero confidence, which is the kraken2 default settings, the distribution was 95.73% Bacteria, 2.38% Archaea, 1.76% Eukaryota, and 0.13% viruses. For the taxa that were present at greater than 0.01% abundance with a confidence flag of “0.9”, the distribution was 97.0% Bacteria, 3.0% Archaea, and 0.04% Eukaryota. The following results are all based on the confidence of “0.9”. There were 12 bacterial phyla identified and 146 families. The predominant bacteria phyla were *Bacteroidetes* (42.7%), *Firmicutes* (24.1%), *Proteobacteria* (7.9%), *Actinobacteria* (5.4%), and *Fibrobacteres* (5.2%). Within *Bacteroidetes* the most predominant family was *Prevotellaceae* which made up 83.9% of the bacteria found in this phylum. Within *Firmicutes* the predominant families were *Lactobacillaceae*, *Lachnospiraceae*, *Ruminococcaceae*, *Eubacteriaceae*, *Selenomonadaceae*, and *Leuconostocaceae* in descending order of presence and they constituted a total of 79.2% of the families in this phylum. The predominant families within *Proteobacteria* in descending order from most present were *Moraxellaceae*, *Pseudomonadaceae*, *Xanthomonadaceae*, *Enterobacteriaceae*, *Burkholderiaceae*, *Desulfovibrionaceae*, *Vibrionaceae*, *Acetobacteraceae*, and *Aeromonadaceae* making up 45.1% of the families in this phylum. For *Actinobacteria*, the predominant families were *Atopobiaceae*, *Bifidobacteriaceae*, and *Eggerthellaceae* in descending order and they made up 70.7% of the families in this phylum. For the *Fibrobacteres* phylum, the predominant family was *Fibrobacteraceae* which made up 100% of this phylum. The percentage of phylum reads classified at the species level was 82.1% with 334 total species classified.

**Fig 2:**
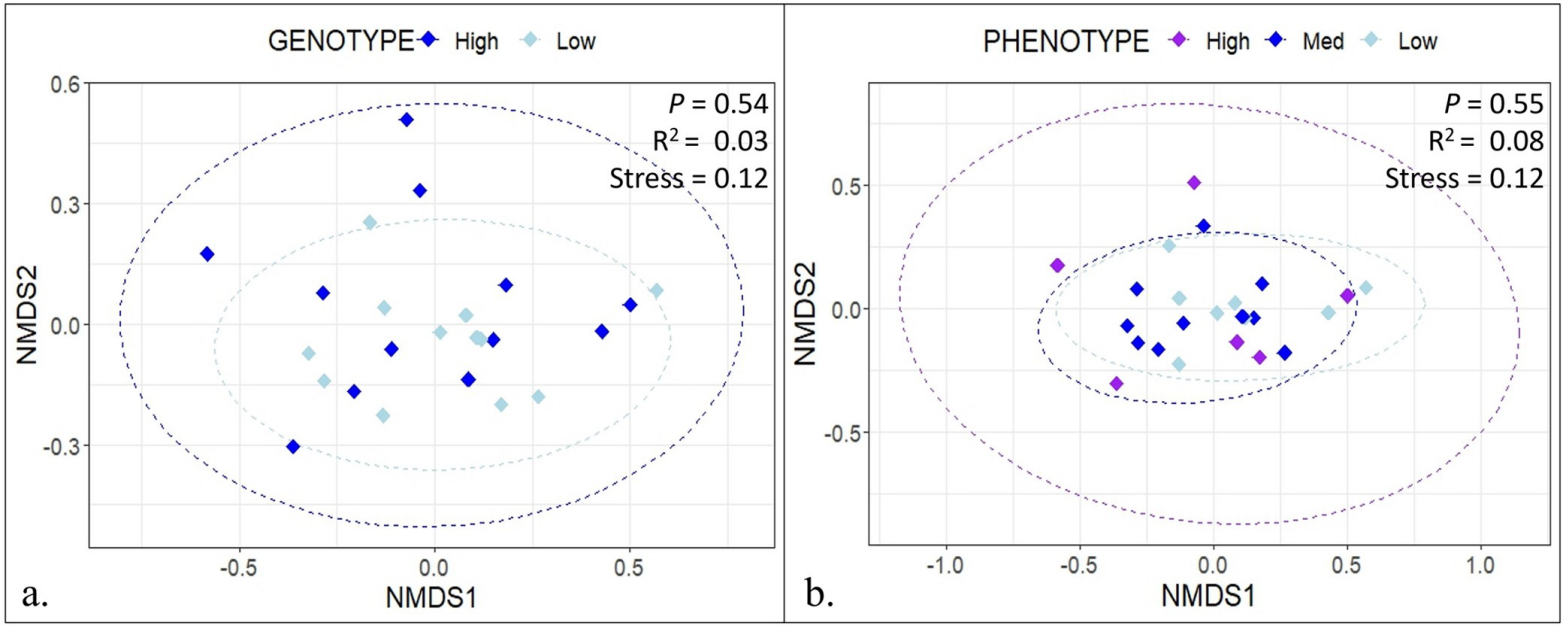
NMDS plot comparing rumen microbiome of cows with different milk production. Non-metric multidimensional scaling (NMDS) plots based on the Bray Curtis dissimilarity between the samples comparing the rumen content of cows **a** with high versus low milk production based on genomic predicted transmitting ability for milk (GPTAM) and **b** cows with high, medium, and low milk production based on phenotypes. The 95% confidence intervals fitted into the spatial ordination are indicated by ellipses.

**Fig 3:**
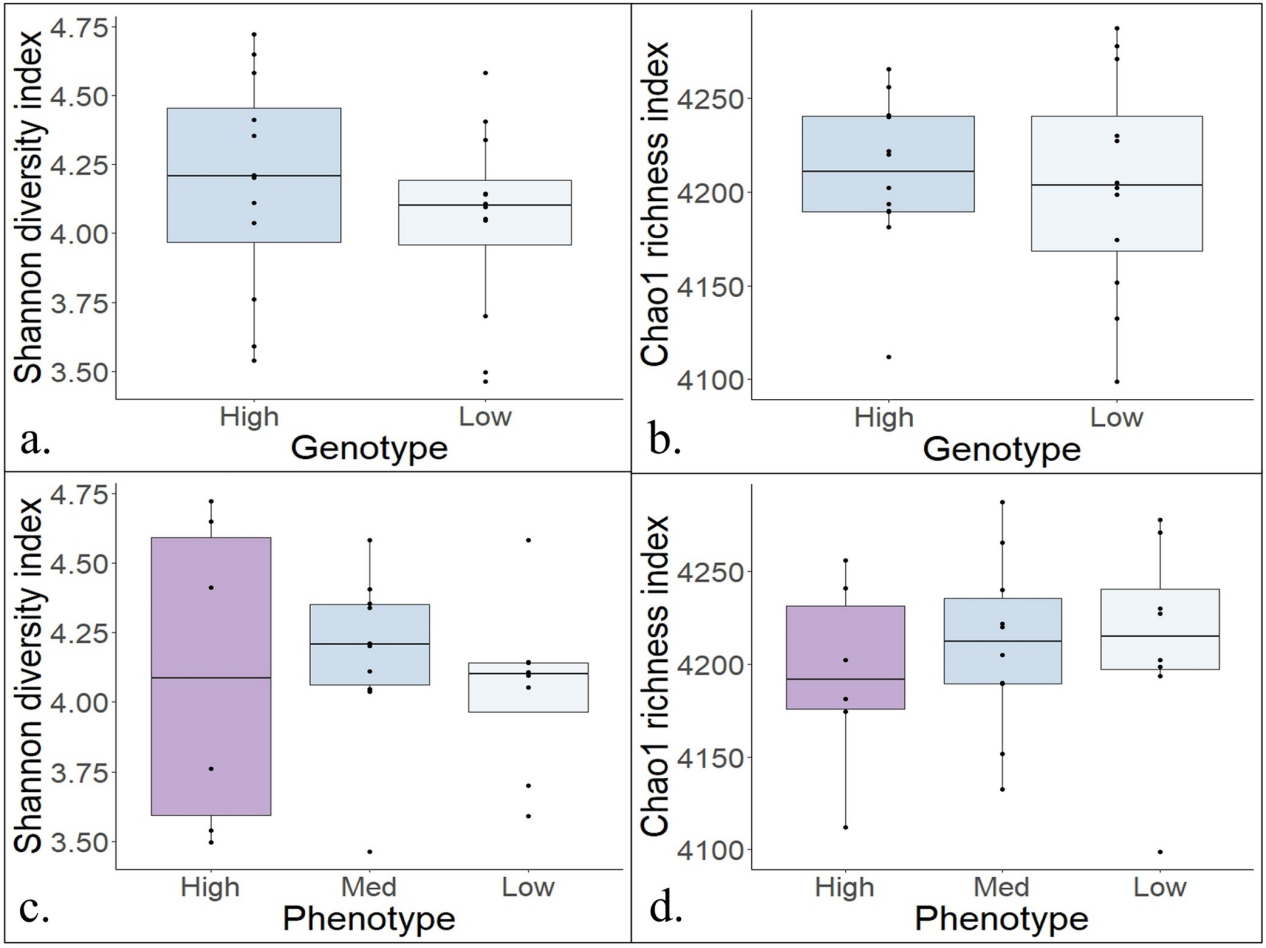
Alpha diversity of rumen microbiome of cows with different milk production. Alpha diversity of rumen content microbiome of cows with high versus low milk production based on genomic predicted transmitting ability for milk (GPTAM, **a, b**). GPTAM: high n = 12 and low n = 12. *P*-value of Student’s *t*-test. Cows with high, medium, and low milk production based on phenotypes (**c, d**). Phenotype based on observable milk production: high n = 6, medium n = 10, and low n = 8. *P*-value of one-way ANOVA.

Based on the Bray Curtis dissimilarity depicted in the NMDS plots in Fig 2, there was no separation of samples when comparing the GPTAM high versus low production groups (*P* = 0.54). Additionally, no separation was observed when comparing the phenotypic high, medium, or low production groups (*P* = 0.55). There were also no observed differences in the alpha diversity of the samples in the GPTAM or in the phenotype comparison (Fig 3). Moreover, the dataset was agglomerated at the levels of phylum, class, order, family, genus, and species and the differential abundance of OTUs was tested, there were no OTUs found to be differentially abundant in the genotype or phenotype comparison.

### Canonical correspondence analysis plot

**Fig 4:**
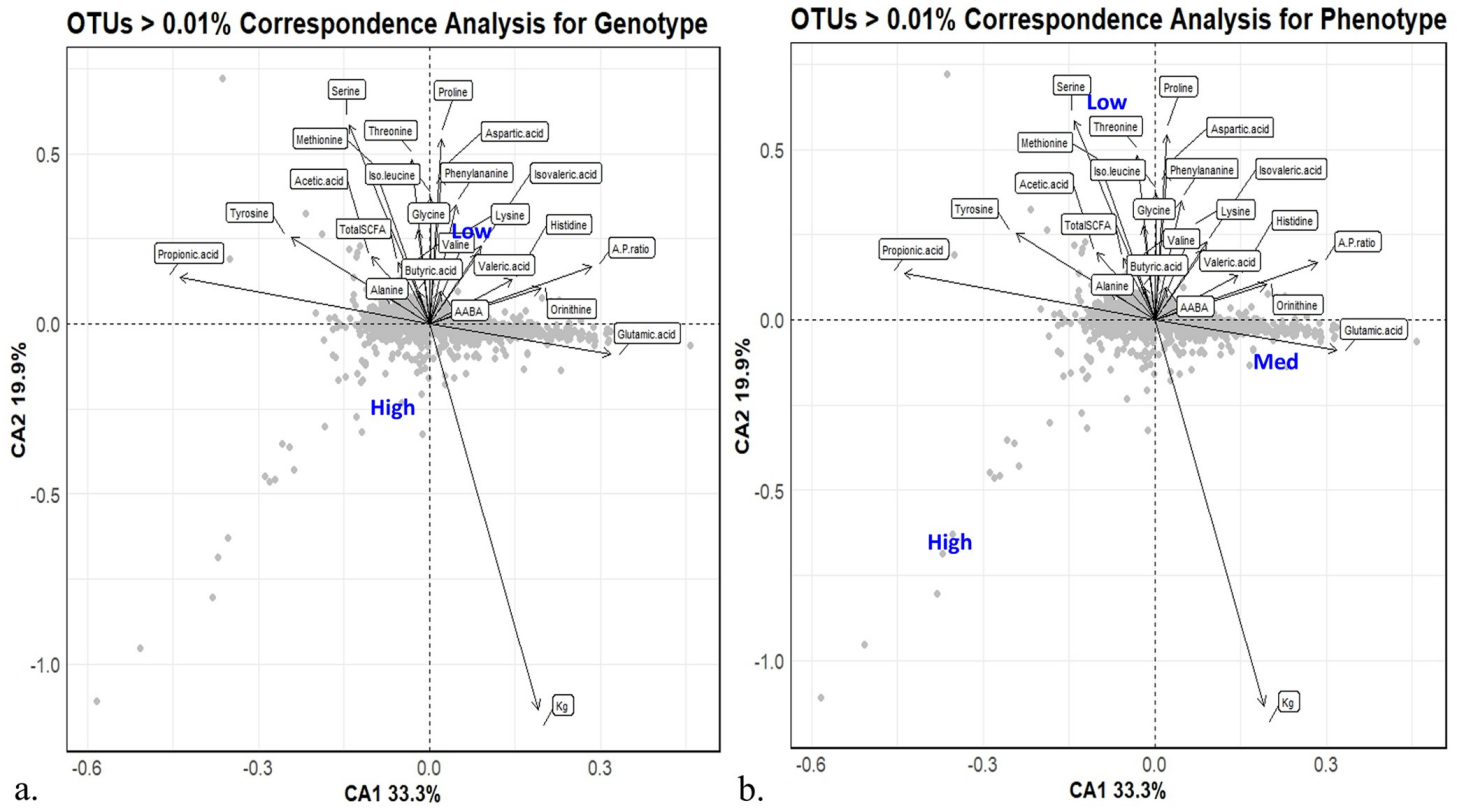
CCA plot depicting microbes and metabolites of the rumen in cows with different milk production. The correspondence analysis plot depicting the square root transformed relative abundances and the metabolite concentrations for the rumen content of cows (**a**) with high versus low milk production based on genomic predicted transmitting ability for milk (GPTAM) and (**b**) cows with high, medium, and low milk production based on phenotypes.

The canonical correspondence analysis (CCA) plot was created to relate the abundance of bacterial populations to the environmental variables for the GPTAM and phenotype comparison (Fig 4). The variance explained by the dimensions was 33.3% for dimension 1 (x-axis) and 19.9% for dimension 2 (y-axis). It can be observed that milk produced (kg), propionic acid concentration in the rumen content, and serine and proline concentrations in the rumen content are the most influential environmental variables. Additionally, it is observed that when plotting the phenotype comparison, the medium group in phenotype seems to be closer to the high group in the genotype CCA plot. This could potentially be correlated to more cows in the medium group (n = 10) deriving from the high genotype group (n = 6) compared to the low genotype group (n = 4).

### Pearson correlations

**Fig 5:**
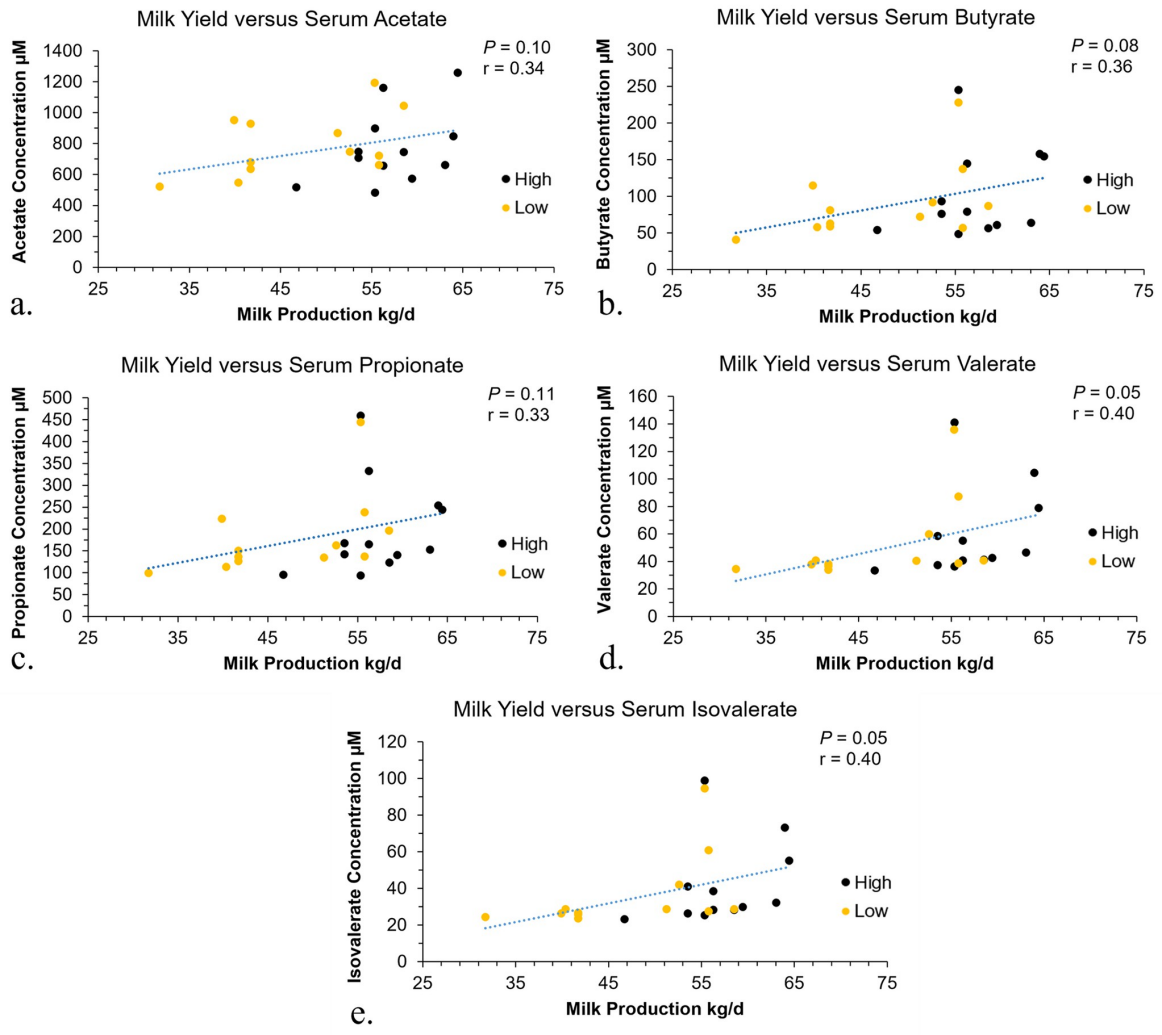
Pearson correlation for blood serum concentrations and GPTAM milk production. Pearson correlations for the serum metabolites by genomic predicted transmitting ability for milk (GPTAM) groups in relation to milk production in dairy cows. The Pearson correlation between milk production in kg and serum acetate concentration in μM (a). The Pearson correlation between milk production in kg and serum butyrate concentration in μM (b). The Pearson correlation between milk production in kg and serum propionate concentration in μM (c). The Pearson correlation between milk production in kg and serum valerate concentration in μM (d). The Pearson correlation between milk production in kg and serum isovalerate concentration in μM (e).

**Fig 6:**
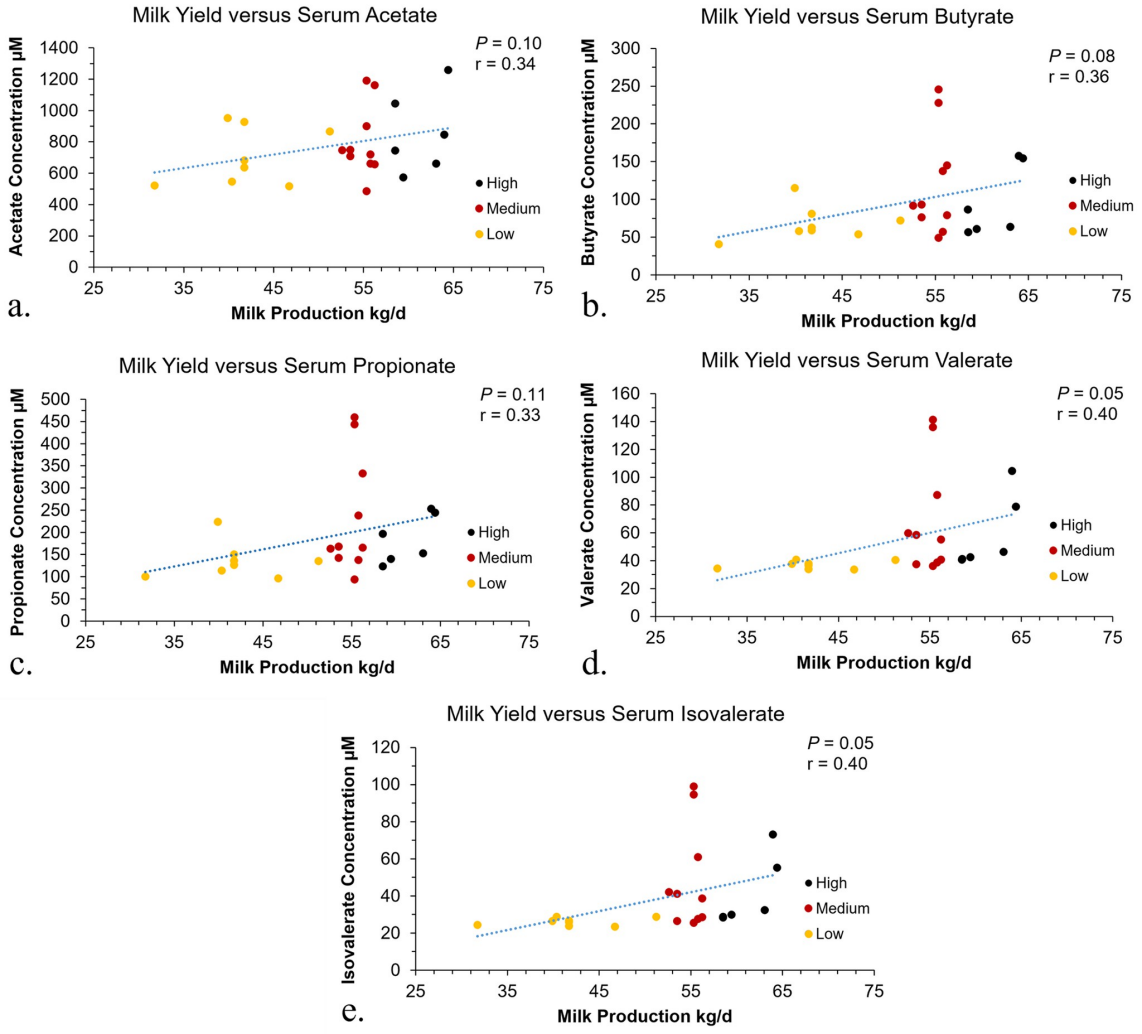
Pearson correlation for blood serum concentrations and phenotype milk production. The Pearson correlations for the serum metabolites by phenotype in relation to milk production in dairy cows. The Pearson correlation between milk production in kg and serum acetate concentration in μM (a). The Pearson correlation between milk production in kg and serum butyrate concentration in μM (b). The Pearson correlation between milk production in kg and serum propionate concentration in μM (c). The Pearson correlation between milk production in kg and serum valerate concentration in μM (d). The Pearson correlation between milk production in kg and serum isovalerate concentration in μM (e).

Pearson correlations were conducted to compare the rumen content and serum metabolite concentrations to milk production. The correlations that were significant were depicted in Fig 5 for GPTAM and Fig 6 for phenotype, these were the correlations between the SCFA concentrations in the serum and milk production. The concentrations of SCFA in the rumen content were not significantly correlated with milk production. The AA concentrations in rumen content or serum did not have significant correlations to the milk production.

## Discussion

Being able to characterize differences between the high and low producing dairy cows might enable the development of strategies to help the population move towards greater milk production. Considering the role of the rumen environment with milk production may move us closer to the use of precision feeding. This is favorable from economic and sustainability perspectives. With increasing availability and cost-effectiveness of metabolome and metagenome analysis it is possible to use these techniques to explore these goals. With these technologies the associations between the ruminal microbiome and metabolome as well as the association of the host metabolome with milk production can be explored.

### Rumen content metabolome

There was no evidence of differences in the rumen content SCFA or AA concentrations or proportions when comparing different milk production groups based on GPTAM or phenotype classification. Contrary to our findings, a study identified 92 rumen metabolites that differed between high versus low producing dairy cows [10]. Furthermore, studies have identified a large number of metabolites that are found in rumen content [7, 25] that can be associated with metabolic disease. It was observed that the methionine concentration in the rumen content of high producing cows was lower than that of the low producing cow. Methionine can be used for microbial protein synthesis by the microbes in the rumen, which would help provide energy for production. Depleted levels of methionine in the high producing cows could be indicative of greater microbial crude protein production in the rumen and availability for milk production; however further studies focused on methionine in high versus low producing cows would be needed to confirm this.

A limitation of this study was that a targeted analysis was conducted for specific metabolites. An untargeted analysis considering more metabolites could have captured potential differences more successfully [26]. Another limitation was that the exploratory analysis for phenotype was conducted on the same cows that had been previously selected based on their GPTAM, thus they may not be truly representative of the entire population of cows.

### Serum metabolome

Similar to the rumen content metabolome analysis, there were no differences observed in the serum SCFA or AA concentrations or proportion when comparing the high and low producing cows based on GPTAM. Likewise, no differences were observed in the AA concentrations or proportions when comparing the groups by phenotype. However, it was observed that the serum acetate to propionate ratio was smaller in the medium production group compared to the low production group when comparing the two phenotype groups. When considering the proportions of these SCFA, acetate had a greater proportion in the low group compared to the medium production group. Acetate is important for the production of de novo synthesis of fatty acids in milk as it provides carbons and reducing equivalents for lipogenesis pathway [27]. An increase in milk fat yield following a negative quadratic pattern was observed with the infusion of increased concentrations of acetate into the rumen [28]. Although acetate is important for milk and milk fat yield, too high of a concentration or proportion could decrease milk fat yield.

Propionate had a greater proportion in the medium group compared to the low production group. Circulating propionate is the main substrate for gluconeogenesis in the liver of dairy cows [29]. The glucose is then used as substrates for the production of lactose in the mammary gland. Lactose, in turn, draws water into the mammary gland as it is the osmotic regulator in milk [30]. Thus, greater proportions of propionate in the serum indirectly contribute to greater milk production as its downstream substrates are important for milk production. By decreasing the proportion of acetate and increasing the proportion of propionate in the serum, the medium-producing cows experience a boost in milk yield while retaining milk fat yield.

A study considered what metabolites are present in different biofluids [31], and it was observed that lactate, glucose, and acetate are metabolites with the highest concentration in serum in dairy cows. Although the current study did not report glucose as mentioned above propionate is used as a precursor for glucose. There was a numerical difference in propionate concentration when considering the high and medium production phenotype groups. Since the blood from the current study was only collected at one time point, it is possible that propionate in the medium production group may have been converted slower to glucose compared to that of the high producing group. How readily glucose becomes available could affect its use by the body, however, this is out of the scope of the current study.

### Microbial communities

The distribution for the microbial communities followed a similar distribution to that observed in other studies that considered the rumen content of high producing dairy cows, with *Bacteroidetes* and *Firmicutes* being the most predominant phyla [32] making about 82% of the total bacteria found in the rumen content. There were no evidence of differences observed between or within the bacterial communities present in the rumen when comparing GPTAM or phenotype groups. There was also no difference observed in the relative abundances of the microbial communities. Although it is possible to have the same metabolite profile with different microbes present, for this study there were no differences observed in the rumen metabolite profile or microbial communities. When another study compared rumen content of Holstein cows that were high versus low producing based on milk protein yield, [11] it was observed that *Protovella* species to be more abundant in the cows that were high producing, and these cows also had less abundance of methanogen organisms. Using GPTAM to group cows, instead of a physically measured attribute to separate the cows into low versus high producing could partly attribute to the lack of differences observed in the current experiment. By using a phenotypical attribute there is greater certainty that all cows in a group are either high or low producing. Another potential reason may be that the average production for milk yield that characterized high producing (37.2 kg/d) in previous reports [11] was lower than the average production for the low group in the current study (47.2 kg/d).

A study was conducted comparing the rumen microbiome of high and low-producing Holstein cows [12]. It was observed that several *Archaea*, *Bacteria*, *Eukaryota*, *and Viruses* differed between high versus low producing dairy cows using rumen metagenomics. Unfortunately, in the current studies no differences were observed. The use of a different database for classification and confidence settings may play a part in different results observed across studies. A study focusing on the rumen microbial genomes observed that the Refseq database classified only about 50% of reads [33]. However, it is worth noting that the average milk yield for the low-producing group in our study was still greater than the average milk yield (44.57 kg/d) reported by others for their high-producing group [12]. Additionally, the two groups considered in the study had a difference of milk production of almost 20 kg/d [12], for the current study the groups only had a difference in milk production of about 10 kg/d when comparing the high versus low production by GPTAM. Differences in microbial relative abundances of some bacteria such as *Proteobacteria* were observed in another study comparing rumen microbial communities and metabolites of high versus low producing cows [10]. In the mentioned study, the high producing group averaged 39.6 kg of milk which was about 15.8 kg greater than the average milk production (23.7 kg) of the low producing group [10]. Even though the cows in this study were divided into high and low milk production, all of the cows are relatively high producing, thus it is possible that a larger difference in the average milk production between the groups might be necessary to detect differences in the rumen content microbiome. However, it is also possible that more cows were needed per group to find a statistical difference if a difference exists. Finally, it may be that the amount of milk that was considered in the low production groups may not be low enough to observe a difference in the rumen environment.

### CCA plot

The CCA plot is a helpful tool in the identification of correlations between the relative abundances of microbes and environmental variables such as metabolites analyzed from the same samples. An observation from comparing the GPTAM and phenotype CCA plots is that the medium phenotype group seems to be closer related to the high GPTAM group coinciding with most of the medium production cows having been in the high production group in the GPTAM comparison. Additionally, the cows in the medium group have a milk production average that is closer to that of the high group than the low group. Considering that there were no major differences observed for the microbial and metabolite profile for the rumen, no pathway or functional analysis was considered for the dataset.

### Pearson correlations

The Pearson correlations were used to compare the association between rumen and serum metabolites to milk production. There were no significant correlations observed for the rumen content; however, for the serum it was observed that the metabolite concentration for SCFA was positively correlated with milk production and this relationship was visually easier to distinguish based on phenotype characterization compared to GPTAM. Thus, the greater the concentration of SCFA metabolites in the serum the greater the milk production. This may be due to the previously mentioned roles of the SCFA such as acetate and propionate in milk synthesis and production. A previous study has observed that the host metabolome was associated with milk protein yield [11]. Thus, it is possible that post digestive absorption and processes have a stronger association to milk production than the rumen microbiome or metabolome [34]. For example, the capacity of the ruminal epithelium for absorption may be associated with milk production. Another important process to consider is the liver metabolism of substrates that are made available after ruminal absorption of end products. Differences in the liver metabolism of cows may be a factor contributing to differences in milk production. Additionally, feed intake can have a great influence on the performance of dairy cows; however, due to the farm set up in this study, individual feed intake for the cows was not available. As milk production increases interactions with rumen fermentation end products and the host needs to be considered more closely to help better identify factors that influence milk production.

## Conclusions

One way to help address concerns for economic and environmental sustainability in the dairy industry will be to help increase milk production in individual cows. With the use of multi-omic techniques, it is possible to identify associations between the microbiome and metabolome of high versus low producing cows. In the current study, no differences were observed in the microbiome or metabolome of different yielding cows based on GPTAM or phenotype. It was observed that host serum SCFA metabolites were significantly associated with milk production. Thus, future efforts should focus on understanding differences between rumen absorption and post absorption processes of metabolites, such as liver metabolism, when comparing cows that have different milk yields.

## Supporting information

S1 File Raw data file for metabolites and microbiome. Raw data file for metabolite and microbiome of cows with different milk production levels. Individual cow data for kraken analytic matrix, rumen metabolites, and serum metabolites.
(XLSX)
